# Supplementary material for: CRISPR screen decodes SWI/SNF chromatin remodeling complex assembly
Source: Nat Commun. 2025 May 30;16:5011. doi: 10.1038/s41467-025-60424-x (PMC12125367; doi:10.1038/s41467-025-60424-x)
Supplement: Supplementary file 1 — Supplementary Information [file 41467_2025_60424_MOESM1_ESM.pdf]

CRISPR screen decodes SWI/SNF chromatin remodeling complex assembly

Supplementary Information:

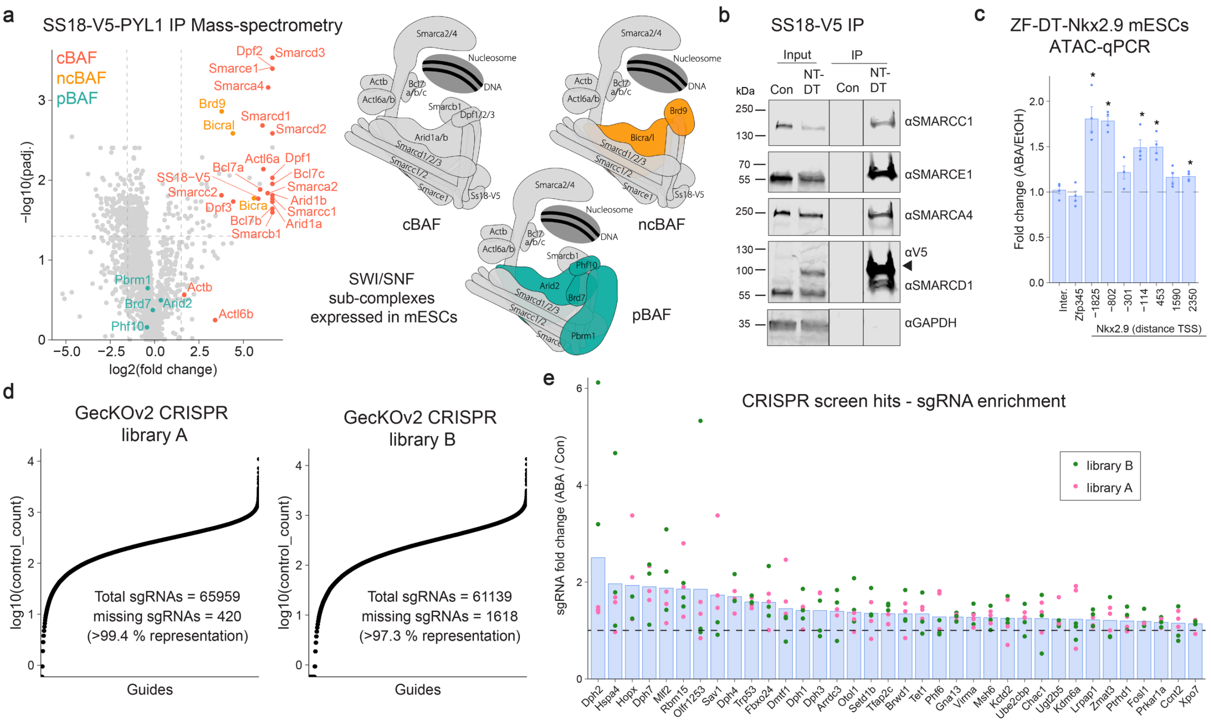

Supplementary Fig. 1 | Related to Figure 1 Genome-wide CRISPR KO screen reveals novel regulators of SWI/SNF activity.

a. Volcano plot showing proteins detected in the SS18-V5-PYL1 immunoprecipitation mass-spectrometry proteomics analysis. Fold change reflects proteins enriched in *ZF-DT-Nkx2.9* mESCs expressing SS18-V5-PYL1 compared to *ZF-DT-Nkx2.9* mESCs not expressing the fusion protein. Highlighted in red are the SWI/SNF (cBAF) subunit proteins; in yellow subunits unique to ncBAF and in green subunits unique to pBAF. Only cBAF and ncBAF complexes are pulled-down via the SS18 subunit. P-value calculated using ANOVA and adjusted with Benjamini Hochberg correction (see Methods).
b. Western blot analysis of SS18-PYL1-V5 immunoprecipitation using V5-trap magnetic beads, shows pull down of SMARCC1, SMARCE1, SMARCA4 and SMARCD1 subunits in *ZF-DT-Nkx2.9* mESCs (NT) but not in the control *ZF-DT-Nkx2.9* mESC that do not express SS18-PYL1-V5 (Con) (n = 1). Arrow indicates height of SS18-V5 fusion protein.
c. ATAC-qPCR shows a gain in chromatin accessibility at *Nkx2.9* locus after ABA induced SWI/SNF-recruitment for 24 hours. qPCR analysis performed using seven primer pairs tilling the *Nkx2.9* locus and three control primers (*Intergenic*, *Zfp345* and *Rpl12*). Fold change normalized to *Rpl12*. Statistical significance was calculated using two-sided t-test compared to *Intergenic* (*significant p-values left to right: p = 0.007; p = 3.5e-5; p= 0.007; p = 0.002; p = 0.028), replicates n = 4.
d. Distribution of sgRNAs in library A and B in control cells after GecKOv2 lentivirus library transduction. Sequencing reveals diverse representation and high coverage of sgRNAs.
e. Fold change of sgRNAs in library A and B. Bars represent the mean fold change between ABA treated and EtOH controls. Depicted genes have MAGeCK Enrichment score < 1.00E-4, except for *Fosl1* (E. score = 1.53E-4), *Kdm6a* (E. score = 5.99E-3) and *Setd1b* (E. score = 2.96E-4). n = 6 sgRNAs targeting all mouse genes.
Source data are provided as a Source Data file.

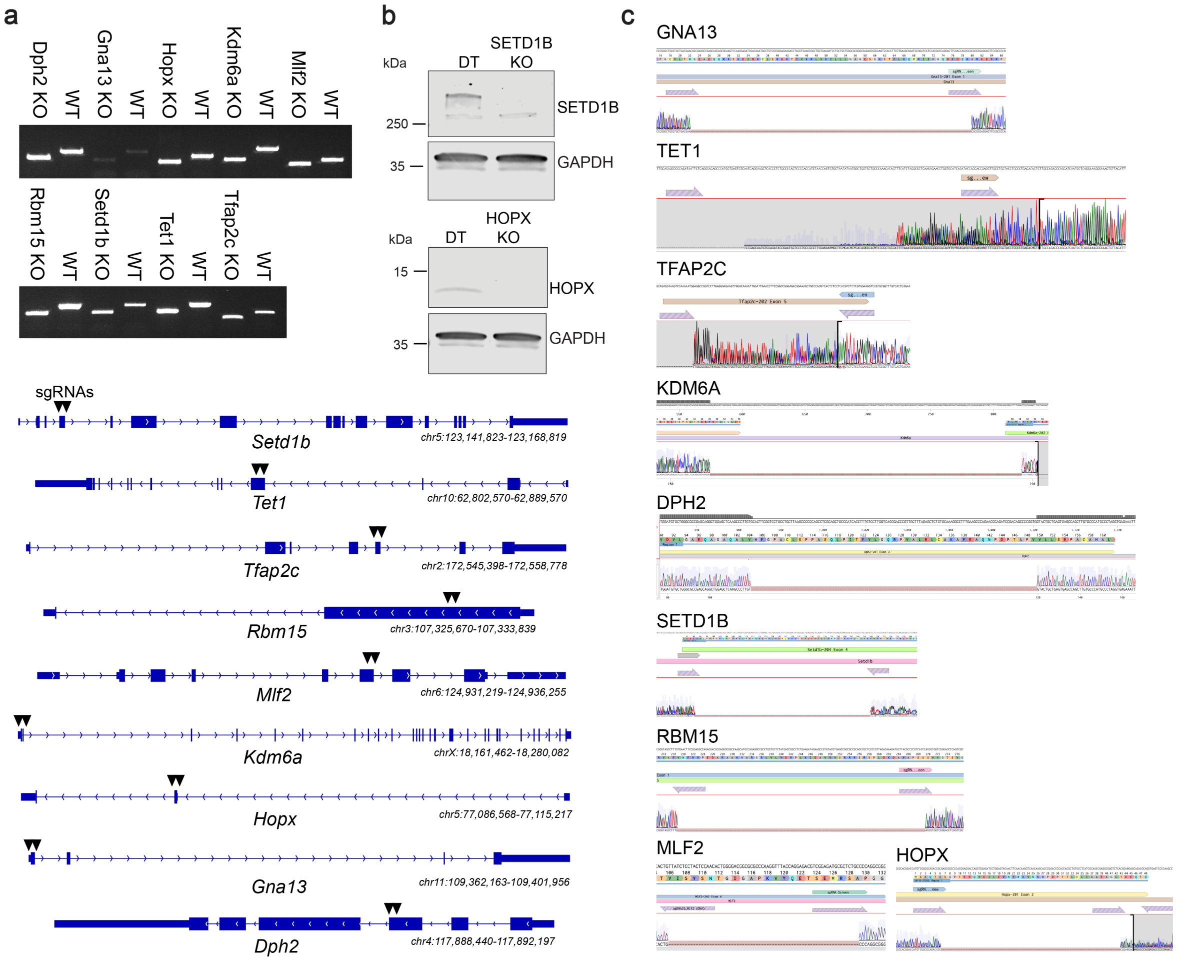

Supplementary Fig. 2 | Related to Figure 2 Validation of top hits from genetic screen in mESCs.

a. Genotyping PCRs of CRISPR KO mESC lines compared to WT mESCs. Shifted bands indicate successful KO of the target locus. Below, schematic of *Dph2*, *Mlf2*, *Rbm15*, *Hopx*, *Tfap2c*, *Gna13*, *Setd1b*, *Kdm6a* and *Tet1* genomic loci in mouse genome (mm10); black arrows indicate exons targeted by pairs of gRNAs to KO individual genes.
b. Western blot analysis of SETD1B and HOPX KO mESC clones (n = 1). Antibodies ordered for TET1, KDM6A and DPH2 did not work. GAPDH as loading control.
c. Sanger sequencing analysis shows KO by indel/frameshift of all 9 genes in individual mESC clones.
Source data are provided as a Source Data file.

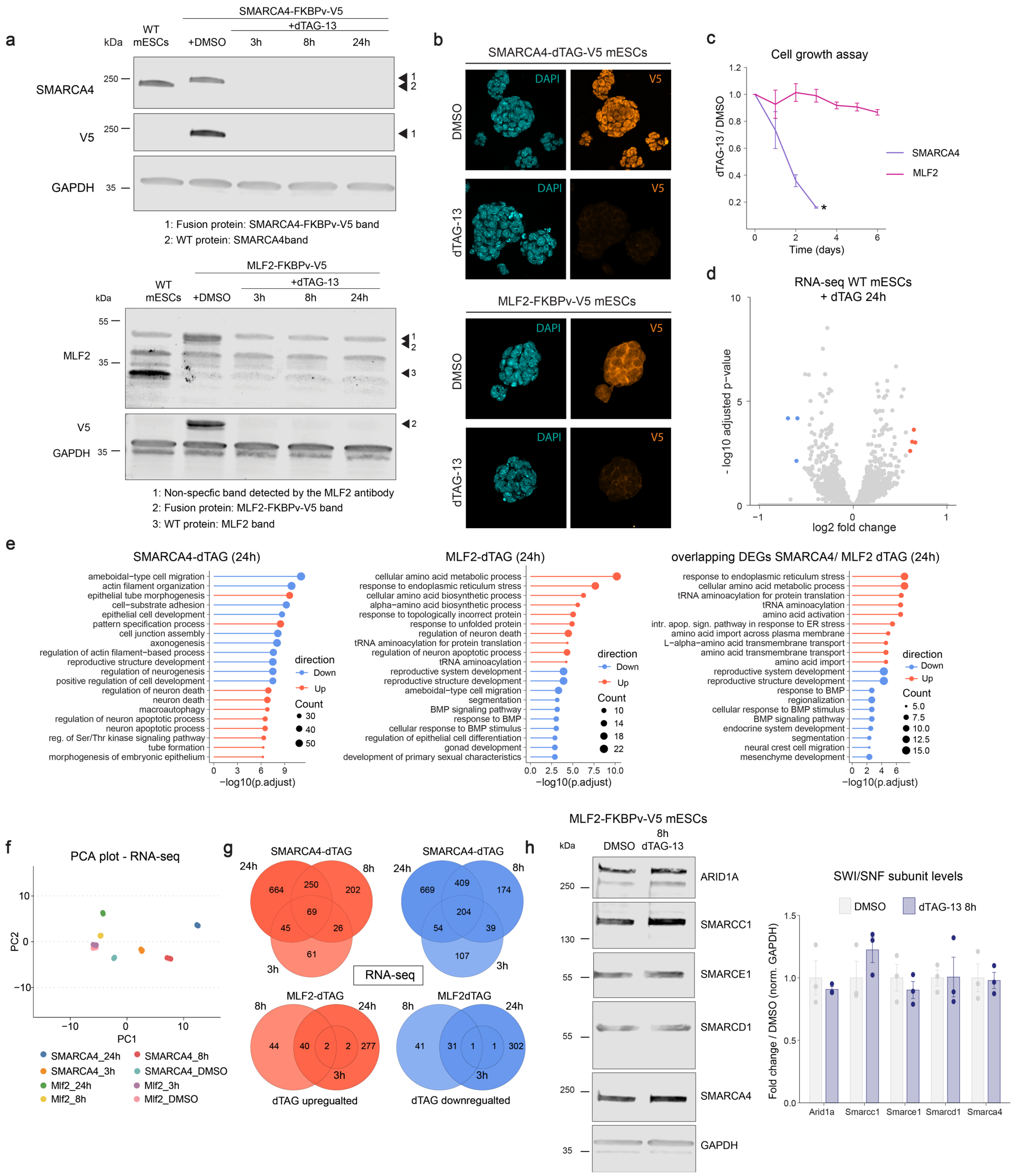

Supplementary Fig. 3 | Related to Figure 3 MLF2 regulates a subset of SWI/SNF target genes in mESCs.

a. Top: Western blot analysis of SMARCA4 levels in SMARCA4-dTAG mESCs treated with DMSO, 3h, 8h and 24h with dTAG-13 compared to WT mESCs. GAPDH as loading control (n=1). Bottom: Western blot analysis of MLF2 and V5 levels in MLF2-dTAG mESCs treated with DMSO, 3h, 8h and 24h with dTAG-13 compare to WT mESCs. GAPDH as loading control (n = 1). Note: MLF2 antibody detects a non-specific band just above the MLF2-FKBPv-V5 band.
b. Confocal images of SMARCA4 and MLF2 dTAG mESCs after 24h of dTAG-13 or DMSO treatment. V5 antibodies used to visualize tagged proteins.
c. Growth curve of MLF2-dTAG and SMARCA4-dTAG mESCs treated with dTAG-13. Replicates n = 3, * indicates complete cell death.
d. Volcano plot shows only 7 DEGs in WT mESC after 24h of dTAG-13 treatment compared to DMSO controls. Significantly downregulated genes are colored in blue and upregulated in red (sig. = padj. < 0.05 & |FC| > 1.5; replicates n = 3). Adjusted p-values were calculated using the Benjamini-Hochberg correcting using DESeq2 software, see Methods.
e. GO-terms of DEGs from SMARCA4 dTAG and MLF2 dTAG cells after 24h of dTAG-13 treatment.

f. PCA plot of RNA-seq samples from MLF2-dTAG and SMARCA4-dTAG mESCs treated with dTAG13 (3h, 8h, 24h) or DMSO. Replicates n = 3.
g. Venn diagrams showing overlaps of DEGs from RNA-seq at 3h, 8h and 24h of dTAG-13 treatment in MLF2 and SMARCA4 dTAG treated mESCs. Upregualted genes are shown in red and downregulated genes are shown in blue.
h. Left: Western blot analysis of SWI/SNF subunit levels in MLF2 dTAG mESCs, treated with 8h of dTAG-13 or DMSO (n=3). Right: Graph shows quantification of replicates normalized to GAPDH (n=3). Significance calculated using two-sided t-test. Source data are provided as a Source Data file.

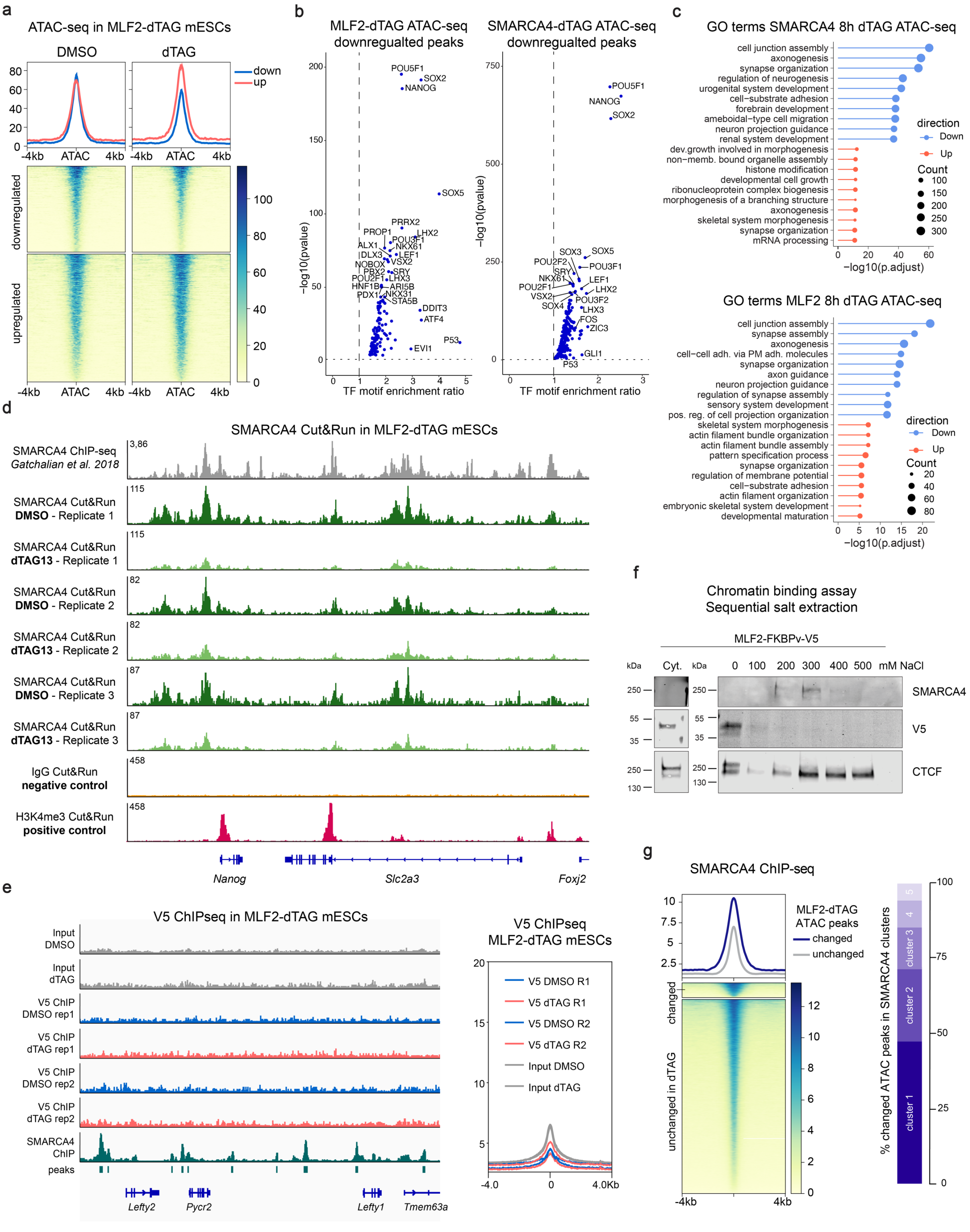

Supplementary Fig. 4 | Related to Figure 4 MLF2 regulates SWI/SNF chromatin remodeling activity in mESCs.

a. ATAC-seq signal at upregulated and downregulated peaks in MLF2-dTAG mESCs treated with dTAG-13 for 8h compared to DMSO control condition (sig. = padj. < 0.05 & |FC| > 1.5; replicates n = 3). Adjusted p-values were calculated using the Benjamini-Hochberg correcting using DESeq2 software, see Methods.
b. Motif analysis of downregulated ATAC peaks at 8h of dTAG-13 treatment in SMARCA4 and MLF2 dTAG mESCs.
c. GO-terms of genes associated to changed ATAC-seq peaks in SMARCA4-dTAG and MLF2-dTAG mESCs after 8h of dTAG-13 treatment.

d. Genome browser tracks depicting SMARCA4 Cut&Run signal for each replicate (n=3) at the *Nanog* locus in MLF2-dTAG mESCs treated with DMSO or dTAG-13 for 8h. IgG negative control and H3K4me3 positive control Cut&Run assays were performed together with the SMARCA4 Cut&Run assays. For comparison with published SMARCA4 ChIP-seq data the genome browser tracks from Gatchalian et al. [49] are included above.
e. Genome browser tracks depicting V5 ChIP-seq and input signal in MLF2-dTAG mESCs compared to published SMARCA4 ChIP-seq[47] peaks at the *Lefty1* locus (left). V5 ChIP-seq signal compared to input signal shows no enrichment and suggests MLF2 does not bind chromatin (right).
f. Sequential salt extraction assay of chromatin bound proteins isolated from MLF2 dTAG mESCs. Cytoplasmic (Cyt.), nuclear and chromatin bound fractions eluted at indicated salt concentrations were loaded for western blot analysis (n=2). MLF2 is localized to the cytoplasm and nucleus but not chromatin bound fractions. As a control we detected CTCF in all fractions, and in particular at high salt concentrations, consistent with its high affinity for chromatin.
g. Left: SMARCA4 ChIP-seq signal at changed and unchanged ATAC peaks in MLF2-dTAG mESCs after 8h of dTAG-13 treatment, ranked according to read density. Right: SMARCA4 ChIP-seq data[47] was clustered into 5 clusters with equal numbers of peaks, ranging from high occupancy (1) to low occupancy (5). Graph shows the % of changed ATAC-seq peaks in MLF2-dTAG mESCs in each cluster.
Source data are provided as a Source Data file.

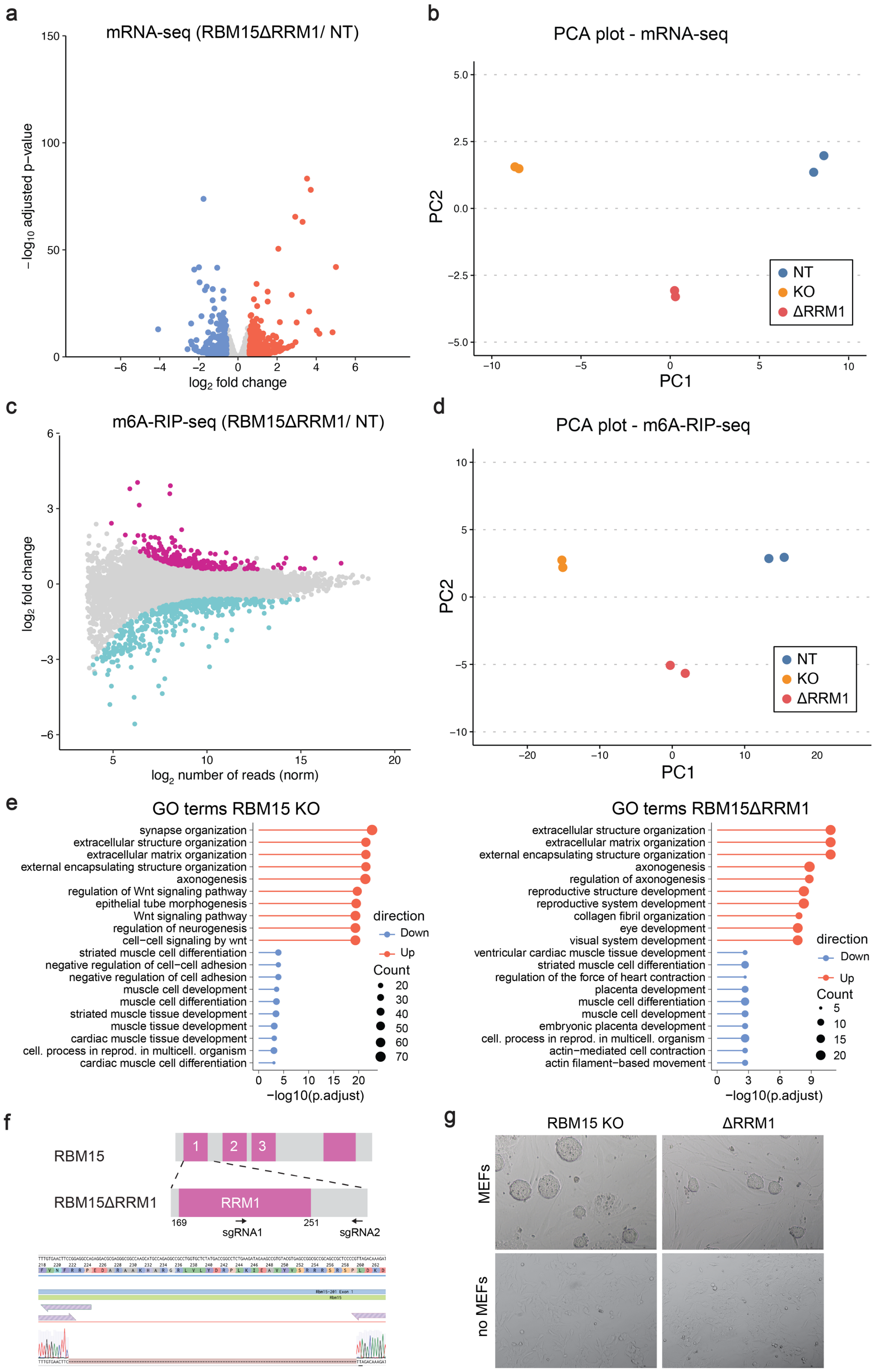

Supplementary Fig. 5 | Related to Figure 5 RBM15 controls m⁶A methylation on specific SWI/SNF mRNAs.

a. Volcano plot showing differentially expressed genes (DEGs) between NT and RBM15 ΔRRM1 mutant mESCs (n=2, FDR < 0.05 and |FC| > 1.5). The 265 downregulated genes are labeled in blue, the 253 upregulated genes are in red.
b. Plot shows principal component analysis (PCA) for RNA-seq from NT, RBM15 KO and ΔRRM1 mESCs.

c. MA plot showing differentially m6A modified mRNA peaks between NT and RBM15 ΔRRM1 mutant mESCs (n=2, FDR < 0.05 and |FC| > 1.5). The 232 downregulated peaks are labeled in cyan, the 423 upregulated peaks are in magenta.
d. Plot shows principal component analysis (PCA) for m6A-RIP-seq from NT, RBM15 KO and ΔRRM1 mESCs.
e. GO-term analysis of up- and down-regulated genes in RBM15 KO and ΔRRM1 mESCs.
f. Schematic of *Rbm15* ΔRRM1 disruption strategy in mESCs; black arrows indicate pairs of sgRNAs used to target RRM1. Sanger sequencing analysis confirms in-frame deletion and disruption of RRM1 domain of RBM15.
g. Images of RBM15 KO and RBM15 ΔRRM1 mESCs grown on MEF-feeders and after extended culture in feeder-free conditions (no MEFs).

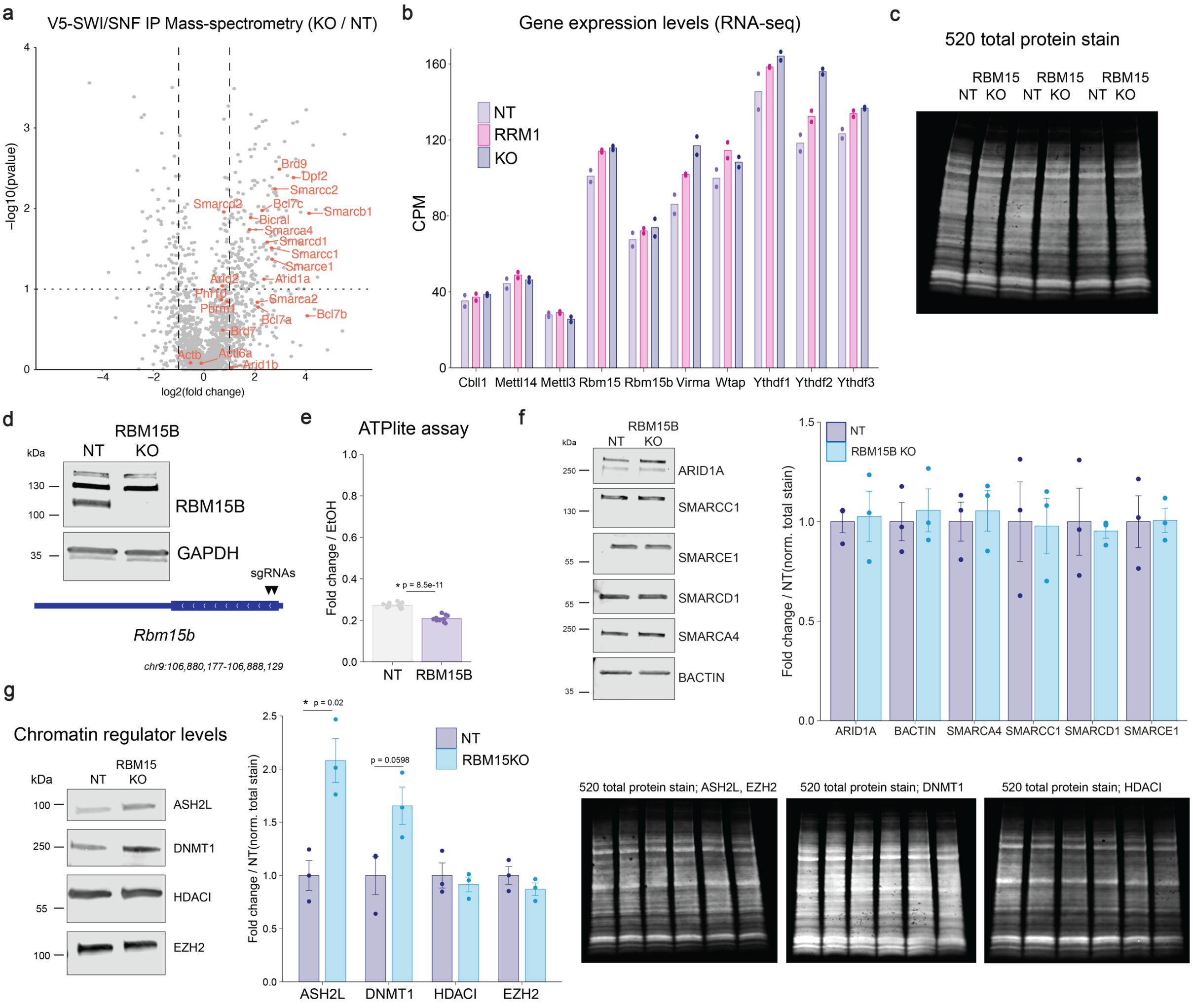

Supplementary Fig. 6 | Related to Figure 6 RBM15 regulates SWI/SNF complex assembly in mESCs.

a. Volcano plot showing all proteins detected in the V5-SWI/SNF IP-MS proteomics analysis. Fold change reflects protein enriched in RBM15 KO mESCs compared to NT mESCs. Highlighted in red are the SWI/SNF subunit proteins. P-values were calculated using ANOVA testing.
b. RNA-seq counts for genes in the m6A RNA methylation pathway in NT, RBM15 KO and ΔRRM1 mESCs (n = 2).
c. Total protein stain of western blot membrane used for quantification, shows equal loading of protein lysates for analysis of SWI/SNF subunit levels in NT, RBM15 KO and ΔRRM1 mESCs.
d. Top, Western blot confirms KO of RBM15B (n=1). Below, schematic of the *Rbm15b* locus in the mouse genome (mm10); black arrows indicate exons targeted by pairs of sgRNAs to KO individual genes.
e. Plot shows ATPlite cell viability assay results following 48h ABA treatment in NT control and Rbm15 *KO ZF-DT-Nkx2-9* mESCs.; replicates n = 12; Fold change values are depicted as mean ± SEM.
f. Left, Western blot analysis of SWI/SNF subunits in RBM15B KO and NT *ZF-DT-Nkx2-9* mESCs (n=3). Right, Graph shows quantification of replicates normalized to total protein stain (n=3). Significance calculated using two-sided t-test. Fold change values are depicted as mean ± SEM.
g. Left, Western blot analysis of chromatin regulators ASH2L, DNMT1, HDAC1 and EZH2 in RBM15 KO and NT mESCs (n=3). Center, Graph shows quantification of replicates normalized to total protein stain (n=3). Significance calculated using two-sided t-test (* = $p < 0.05$). Fold change values are depicted as mean ± SEM. Right, Total protein stain used for quantification of western blot membrane shows equal loading of protein lysates for analysis of SWI/SNF subunit levels in NT and RBM15 KO mESCs.
Source data are provided as a Source Data file.

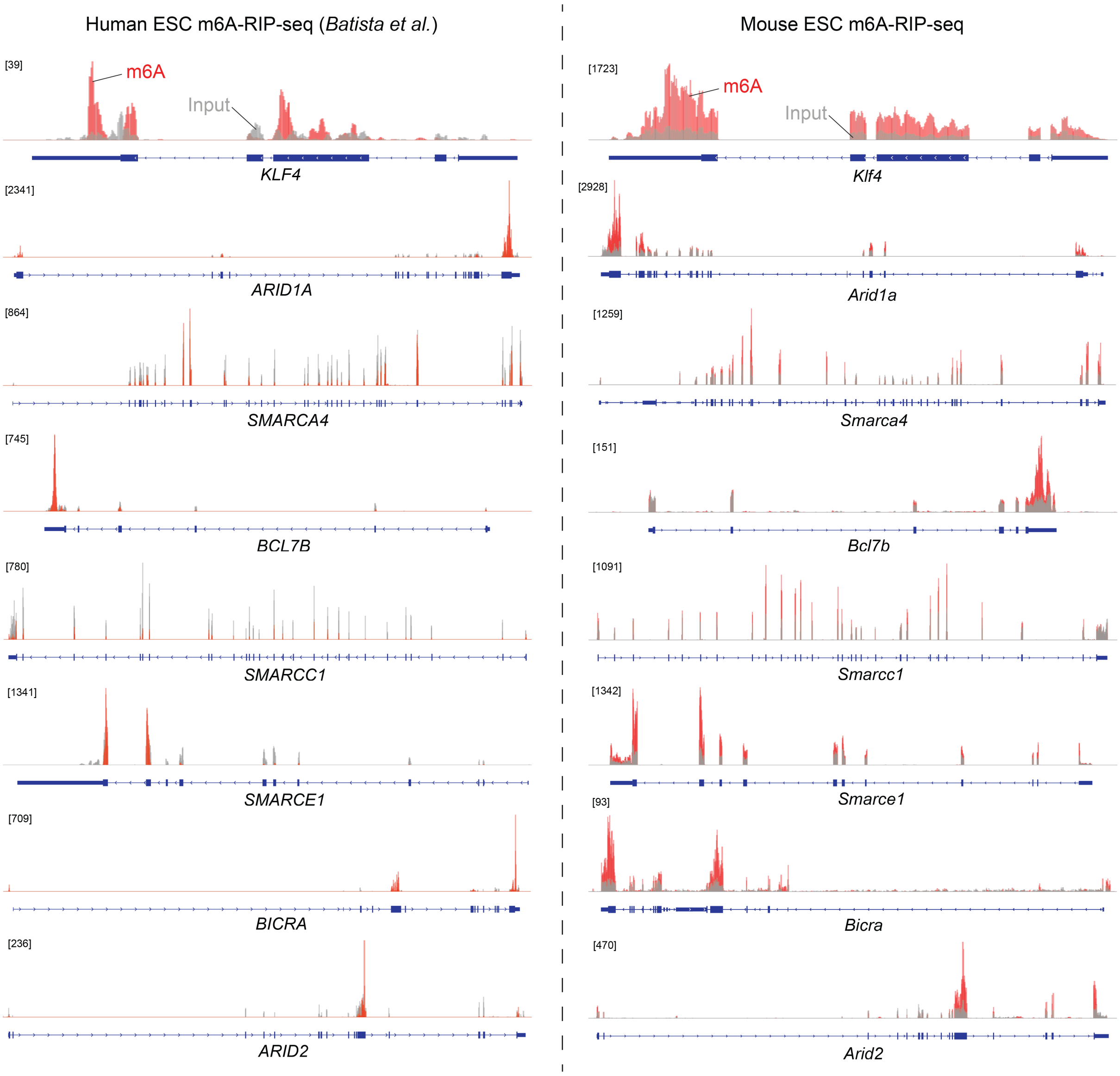

Supplementary figure 7 | Related to Discussion

Genome browser tracks of m^{6}A-RIP-seq data from human and mouse ES cells. The human ES cells data was obtained from a published study by Batista et al.[55] For comparison, the SWI/SNF subunits ARID1A, SMARCA4, BCL7B, SMARCC1, SMARCE1, BICRA and ARID2, as well as the pluripotency gene KLF4, are shown.
