# Supplementary material for: CRISPR screen decodes SWI/SNF chromatin remodeling complex assembly
Source: Nat Commun. 2025 May 30;16:5011. doi: 10.1038/s41467-025-60424-x (PMC12125367; doi:10.1038/s41467-025-60424-x)
Supplement: Supplementary file 2 — Description of Additional Supplementary Files [file 41467_2025_60424_MOESM2_ESM.pdf]

Description of Additional Supplementary Files

Supplementary Data 1. MAGeCK analysis of enrichment scores for all mouse genes in CRISPR KO screen of *DT-ZF-Nkx2.9* mESCs. P-values calculated with permutation test using MAGeCK software, see Methods.

Supplementary Data 2. DESeq2 analysis of differentially expressed genes from RNA-seq experiments in MLF2-dTAG and SMARCA4-dTAG mESCs treated with dTAG13 for 3h, 8h and 24h (Adjusted p-values were calculated using the Benjamini-Hochberg correcting, see Methods).

Supplementary Data 3. DESeq2 analysis of differentially accessible peaks from ATAC-seq experiments in MLF2-dTAG and SMARCA4-dTAG mESCs treated with dTAG13 for 8h (Adjusted p-values were calculated using the Benjamini-Hochberg correcting, see Methods).

Supplementary Data 4. DESeq2 analysis of differentially expressed genes and differential m^6A peaks from RNA-seq and m^6A-RIP-seq experiments in NT, RBM15 KO and ΔRRM1 mESCs (Adjusted p-values were calculated using the Benjamini-Hochberg correcting, see Methods).

Supplementary Data 5. Peptide counts and statistical analysis from SWI/SNF IP-MS analysis of NT and Control mESCs, as well as NT and RBM15 KO mESCs. P-values were calculated using ANOVA testing and adjusted with Benjamini Hochberg correction.

Supplementary Data 6. List of primers and antibodies used in this study.
