# Supplementary material for: CRISPR screen decodes SWI/SNF chromatin remodeling complex assembly
Source: Nat Commun. 2025 May 30;16:5011. doi: 10.1038/s41467-025-60424-x (PMC12125367; doi:10.1038/s41467-025-60424-x)
Supplement: Supplementary file 9 — Reporting Summary [file 41467_2025_60424_MOESM9_ESM.pdf]

nature portfolio

Corresponding author(s): Simon Braun

Last updated by author(s): Aug 1, 2024

Reporting Summary

Nature Portfolio wishes to improve the reproducibility of the work that we publish. This form provides structure for consistency and transparency in reporting. For further information on Nature Portfolio policies, see our Editorial Policies and the Editorial Policy Checklist.

Statistics

For all statistical analyses, confirm that the following items are present in the figure legend, table legend, main text, or Methods section.

n/a	Confirmed	
☐	☒	The exact sample size (*n*) for each experimental group/condition, given as a discrete number and unit of measurement
☐	☒	A statement on whether measurements were taken from distinct samples or whether the same sample was measured repeatedly
☐	☒	The statistical test(s) used AND whether they are one- or two-sided<br>*Only common tests should be described solely by name; describe more complex techniques in the Methods section.*
☒	☐	A description of all covariates tested
☐	☒	A description of any assumptions or corrections, such as tests of normality and adjustment for multiple comparisons
☐	☒	A full description of the statistical parameters including central tendency (e.g. means) or other basic estimates (e.g. regression coefficient) AND variation (e.g. standard deviation) or associated estimates of uncertainty (e.g. confidence intervals)
☐	☒	For null hypothesis testing, the test statistic (e.g. *F*, *t*, *r*) with confidence intervals, effect sizes, degrees of freedom and *P* value noted<br>*Give P values as exact values whenever suitable.*
☒	☐	For Bayesian analysis, information on the choice of priors and Markov chain Monte Carlo settings
☒	☐	For hierarchical and complex designs, identification of the appropriate level for tests and full reporting of outcomes
☐	☒	Estimates of effect sizes (e.g. Cohen's *d*, Pearson's *r*), indicating how they were calculated

Our web collection on statistics for biologists contains articles on many of the points above.

Software and code

Policy information about availability of computer code

Data collection

No commercial, open source or custom code was used to collect the data in this study.

Data analysis

CRISPR screen MAGeCK analysis:
To map reads to corresponding sgRNAs and target genes we used the MAGeCK30 v0.5.9.5 software and determined significantly enriched genes using default normalization settings. We applied an Enrichment positive score cut off of 1.00E-4 to identify hits. Output data were plotted with ggplot2.

RNA-seq analysis:
Sequencing reads were trimmed using cutadapt73 v3.5 (removal of truseq adapter sequences, polyA sequences, and low-quality reads). Trimmed reads were aligned to the mouse genome (mm10) using STAR74 v2.7.5 (STARoptions, --readFilesCommand zcat --runThreadN 8 --outFilterMultimapNmax 20 --alignSJoverhangMin 8 --alignSJDBoverhangMin 1 --outFilterMismatchNmax 999 --outFilterMismatchNoverLmax 0.6 --alignIntronMin 20 --alignIntronMax 1000000 --alignMatesGapMax 1000000). Gene count matrices were obtained from the aligned reads using featureCounts75 v2.0.3 (-t exon -g gene_id -O -T 4 -s 1 -a GRCm38.102). Differential gene expression analysis between conditions was determined using the R package DESeq276 after pre-filtering genes with low counts (rowSums>10), with significance cut-offs set at FDR < 0.05 and fold change > 1.5 in either direction. Output data were plotted with ggplot2.

m6A-RIP-seq analysis:
Sequencing reads were trimmed using cutadapt 3.5 (adapters -m 20 -O 4 -e 0.2 -p). Reads were aligned to the mm10 genome using STAR v2.7.5 (--readFilesCommand zcat --runThreadN 6 --genomeDir GRCm38.102 --outSAMtype BAM SortedByCoordinate --outSAMunmapped Within --outSAMattributes Standard). m6A peaks enriched relative to input mRNA were called using MACS277 v2.7.1 (callpeak --nomodel --keep-dup all -B -q 0.05 --extsize 100 --shift -50 -g mm -t m6A.bam -c input.bam, filtered for score > 200). Peaks from NT, KO and ΔRRM1

datasets within 100 bp were merged using bedtools v2.30 and the numbers of reads from each replicate overlapping with the peak set was counted using featureCounts v2.0.3 (-T 4 -O -p -a -t exon -g gene_id). Differential m6A peak calls were determined using the R software DESeq2 after pre-filtering peaks with low counts (rowSums>50), with significance cut-offs set at padj < 0.05 and fold change > 1.5 in either direction. Output data were plotted with ggplot2. For IGV genome browser snapshots, average of replicates genome coverage files (bigwig) were generated with deepTools78 3.5.2 (bamCoverage --binSize 10 --normalizeUsing RPKM and bigwigAverage for merging replicates). Genomic features of m6A peaks and the distribution along mRNA transcripts was determined using the R software ChIPSeeker79 v1.32.1.

ATAC-seq analysis:
Sequencing reads were trimmed using cutadapt 3.5 (adapters -m 20 -O 5 -p). Reads were aligned to the mm10 genome using bowtie2 v2.4.4 (--maxins 2000 -p 8 -N 1). Alignments were filtered using samtools v1.12 (view -h -F 1796 -q 20). ATAC peaks were called using MACS2 v2.7.1 (callpeak --format BAMPE --call-summits --shift -75 --extsize 150 --keep-dup all -B --SPMR -g mm -t ATAC.bam). Peaks from all replicates within 1 kb were merged using bedtools v2.30, filtered against the mouse blacklist, and the numbers of reads from each replicate overlapping with the peak set was counted using featureCounts v2.0.3 (-T 4 -O -p -a -t exon -g gene_id). Differential ATAC peak calls were determined using the R software DESeq2 after pre-filtering peaks with low counts (rowSums>100), with significance cut-offs set at FDR < 0.05 and fold change > 1.5 in either direction. Output data were plotted with ggplot2. For IGV genome browser snapshots, average of replicates genome coverage files (bigwig) was generated with deepTools v3.5.2 (bamCoverage --binSize 10 --normalizeUsing RPKM --ignoreForNormalization chrM and bigwigAverage for merging replicates). Genomic features of ATAC peaks were determined using the R software ChIPSeeker v1.32.1. deepTools v3.5.2 was used to produce heat map of published SMARCA4 ChIP-seq mean read density across ATAC. GREAT software was used to annotate ATAC peaks with genes to determine gene ontology terms associated with changes in accessibility.

Cut&Run analysis
Sequencing reads were trimmed using cutadapt 3.5 (adapters -m 20 -O 5 -p). Reads were aligned to the mouse mm10 and E. coli K12 MG1655 genomes using bowtie2 v2.4.4 (-p 8 --very-sensitive --no-unal --no-mixed --no-discordant --dovetail -X 1000). Alignments were filtered using samtools v1.12 (view -h -F 1796 -q 20). Cut&Run peaks in mm10 were called using MACS2 v2.7.1 (callpeak --format BAMPE --nomodel --call-summits --shift -100 --extsize 200 --keep-dup all -B -g mm). Peaks from all replicates within 1 kb were merged using bedtools v2.30. Normalization scaling factors were calculated based on the spiked-in E. coli reads obtained from each replicate. Genome coverage files (bigwig) were generated with deepTools v3.5.2 (bamCoverage --binSize 10 --scaleFactor --normalizeUsing RPKM --ignoreForNormalization chrM ChrUn ChrRandom -p max). For IGV genome browser snapshots, average of replicates genome coverage files (bigwig) was generated with deepTools v3.5.2 (bigwigAverage for merging replicates). deepTools v3.5.2 was used to produce heat maps of SMARCA4 Cut&Run mean read density across Cut&Run and ATAC-seq peaks.

ChIP-seq analysis:
Note: For V5-MLF2 ChIP-seq experiments in mES cells, we did not detect any signal enrichment compared to the inputs and therefore have not uploaded this dataset on the GEO server.
Sequencing reads were trimmed using cutadapt 3.5 (adapters -m 20 -O 5 -p). Reads were aligned to the mm10 genome using bowtie2 v2.4.4 (--sensitive -p 8). Alignments were filtered using samtools80 v1.12 (view -h -F 1796 -q 20). V5-ChIP peaks enriched relative to inputs were called using MACS2 v2.7.1 (callpeak ,--format BAMPE -g mm), however < 100 peaks were called in all replicates, suggesting no enrichment relative to input. For IGV genome browser snapshots, genome coverage files (bigwig) were generated with deepTools 3.5.2 (bamCoverage --binSize 10 –normalizeUsing RPKM).

Proteomics analysis:
Raw data were processed using Proteome Discoverer 2.4 software (Thermo Fisher Scientific). Briefly, spectra were extracted and searched against the Mus musculus reference database (Uniprot, 17109 entries) with the native SS18 protein by sequence replaced the bait SS18-V5 protein sequence (used to purify SWI/SNF and ncSWI/SNF but not PBAF complexes47) and an in-house database of common contaminant using Mascot (Matrix Science, London, UK; version 2.6.2). Trypsin was selected as the enzyme, with one potential missed cleavage. Precursor ion tolerance was set to 10 ppm and fragment ion tolerance to 0.6 Da. Carbamidomethylation of cysteine was specified as fixed modification. Variable amino acid modification was oxidized methionine. Peptide-spectrum matches were validated using Percolator a target FDR of 0.01 and a Delta Cn of 0.5. For label-free quantification, features and chromatographic peaks were detected using the “Minora Feature Detector” Node with the default parameters. PSM and peptides were filtered with a false discovery rate (FDR) of 1%, and then grouped to proteins with again an FDR of 1% (strict) or 5% (relaxed) and using peptides with high confidence level. Low abundance resampling was used for missing value imputation. Both unique and razor peptides were used for quantitation and protein abundances are calculated as the average of the three most abundant distinct peptide group. The abundances were normalized on the “Total Peptide Amount” and then “Protein abundance based” option was selected for protein ratio calculation and associated p-values were calculated with an ANOVA test (individual proteins).

Statistical analysis:
The statistical tests performed are disclosed in the corresponding figure legends. Statistical tests were performed using R.

For manuscripts utilizing custom algorithms or software that are central to the research but not yet described in published literature, software must be made available to editors and reviewers. We strongly encourage code deposition in a community repository (e.g. GitHub). See the Nature Portfolio guidelines for submitting code & software for further information.

Data

Policy information about availability of data

All manuscripts must include a data availability statement. This statement should provide the following information, where applicable:

- Accession codes, unique identifiers, or web links for publicly available datasets
- A description of any restrictions on data availability
- For clinical datasets or third party data, please ensure that the statement adheres to our policy

All data derived from next-generation sequencing assays have been deposited on the GEO server (GSE268206). The mass spectrometry proteomics data have been deposited to the ProteomeXchange Consortium via the PRIDE partner repository with the dataset identifier PXD054392 and PXD061408. Additionally, we analyzed previously published SMARCA4 ChIP-seq from WT mESCs (Gatchalian, J., Malik, S., Ho, J., Lee, D.-S., Kelso, T.W.R., Shokhirev, M.N., Dixon, J.R., and Hargreaves, D.C. (2018)) using bigwig and bed files obtained from the cistrome server. Additionally, we analyzed m6A-RIP-seq data from WT human ESCs (Batista et al., Cell Stem Cell (2014)), using Bigwig files obtained from GEO server. All data are publicly available. All analyses were performed using previously published or developed tools, as

indicated in Methods. Details on the bioinformatics pipeline and scripts used to run these published analysis tools can be found at https://github.com/NLykoskoufis/BraunLabPipeline.

Research involving human participants, their data, or biological material

Policy information about studies with human participants or human data. See also policy information about sex, gender (identity/presentation), and sexual orientation and race, ethnicity and racism.

Reporting on sex and gender	N/A
Reporting on race, ethnicity, or other socially relevant groupings	N/A
Population characteristics	N/A
Recruitment	N/A
Ethics oversight	N/A

Note that full information on the approval of the study protocol must also be provided in the manuscript.

Field-specific reporting

Please select the one below that is the best fit for your research. If you are not sure, read the appropriate sections before making your selection.

☒ Life sciences ☐ Behavioural & social sciences ☐ Ecological, evolutionary & environmental sciences

For a reference copy of the document with all sections, see nature.com/documents/nr-reporting-summary-flat.pdf

Life sciences study design

All studies must disclose on these points even when the disclosure is negative.

Sample size	For all experiments the sample size was decided in advance and is listed in the methods section. RNA-seq was performed in triplicates for each condition and for three timepoints (1 mio cells per condition). ATAC-seq was performed in triplicates for each condition (60,000 cells per condition). SMARCA4 Cut&Run was performed in triplicates using E. coli spike-in for normalization for each condition, we performed H3K4me3 Cut&Run and IgG Cut&Run for positive and negative controls. m6A-RIP-seq was performed in duplicates for three conditions (15 mio cells per condition). The proteomics analysis were performed in triplicates for two conditions (20 mio cells per condition). Cell survival assays were performed in triplicates, cell viability assays were performed with n=6 and quantitative western blots were performed in triplicates.
Data exclusions	No data was excluded for this study.
Replication	For all experiments, replication was performed as indicated above and the changes were assessed using indicated statistical methods.
Randomization	Samples were not randomized. Genotypes and treatments of cells had to be known for downstream processing.
Blinding	Investigators were not blinded for this study however all data was collected and processed uniformly and identically for each experiment.

Reporting for specific materials, systems and methods

We require information from authors about some types of materials, experimental systems and methods used in many studies. Here, indicate whether each material, system or method listed is relevant to your study. If you are not sure if a list item applies to your research, read the appropriate section before selecting a response.

Materials & experimental systems

n/a	Involved in the study	
☐	☒	Antibodies
☐	☒	Eukaryotic cell lines
☒	☐	Palaeontology and archaeology
☒	☐	Animals and other organisms
☒	☐	Clinical data
☒	☐	Dual use research of concern
☒	☐	Plants

Methods

n/a	Involved in the study	
☒	☐	ChIP-seq
☒	☐	Flow cytometry
☒	☐	MRI-based neuroimaging

Antibodies

Antibodies used

Primary Antibodies
Antibody Company Reference number Application Dilution
V5 Abcam ab15828 ChIP 1:100
SMARCC1 Cell Signaling 11956 ChIP 1:100
V5 Invitrogen 46-0705 Western blot 1:5000
GAPDH Sigma G8795 Western blot 1:100.000
GAPDH Santa Cruz sc-32233 Western blot 1:10'000
V5 Invitrogen 46-0705 IF 1:250
SMARCA4 Abcam ab110641 IF 1:250
RBM15 Proteintech 10587-1-AP Western blot 1:1000
B-ACTIN Abcam ab8227 Western blot 1:2000
ARID1A Cell Signalling D2A8U Western blot 1:1000
SMARCC1 Cell Signaling 11956 Western blot 1:1000
SMARCE1 Bethyl a300810a Western blot 1:2000
SMARCD1 Santa Cruz sc-135843 Western blot 1:1000
SMARCA4 Santa Cruz sc-374197 Western blot 1:1000
SETD1B Proteintech 55005-1-AP Western blot 1:1000
HOPX Proteintech 11419-1-AP Western blot 1:1000
MLF2 Proteintech 11835-1-AP Western blot 1:1000
RBM15B Proteintech 22249-1-AP Western blot 1:1000
DNMT1 Active motif 39204 Western blot 1:1000
HDAC1 Abcam ab7028 Western blot 1:1000
EZH2 BD Biosciences 612666 Western blot 1:1000
ASH2L Novus nb600-281 Western blot 1:1000

Secondary Antibodies
Antibody Company Reference number Application Dilution
rabbit IgG 488 Invitrogen A11008 IF 1:1000
mouse IgG 568 invitrogen A11031 IF 1:1000
mouse IgG 680 Licor 925-68070 Western blot 1:10.000
rabbit IgG 800 Licor 925-32211 Western blot 1:10.000
mouse IgG 800 Licor 925-32210 Western blot 1:10.000
rabbit IgG 680 Licor 925-68071 Western blot 1:10.000

Validation

V5 (37 citations) https://www.abcam.com/en-us/products/primary-antibodies/v5-tag-antibody-ab15828
SMARCC1 (72 citations) https://www.cellsignal.com/products/primary-antibodies/smarcc1-baf155-d7f8s-rabbit-mab/11956
V5 (145 citations) https://www.thermofisher.com/antibody/product/V5-Tag-Antibody-clone-SV5-Pk1-Monoclonal/R960-25
GAPDH (>1000 citations) https://www.sigmaaldrich.com/CH/en/product/sigma/g8795
V5 (>1000 citations) https://www.thermofisher.com/antibody/product/V5-Tag-Antibody-clone-SV5-Pk1-Monoclonal/R960-25
SMARCA4 (109 citations) https://www.abcam.com/en-us/products/primary-antibodies/brg1-antibody-epncir111a-ab110641
RBM15 (40 citations) https://www.ptglab.com/products/RBM15-Antibody-10587-1-AP.htm
B-ACTIN (>1000 citations) https://www.abcam.com/en-us/products/primary-antibodies/beta-actin-antibody-ab8227
ARID1A (119 citations) https://www.cellsignal.com/products/primary-antibodies/arid1a-baf250a-d2a8u-rabbit-mab/12354
SMARCE1 (33 citations) https://www.fortislife.com/products/primary-antibodies/rabbit-anti-baf57-smarce1-antibody/BETHYL-A300-810
SMARCD1 (24 citations) https://www.scbt.com/p/baf60a-antibody-23
SMARCA4 (15 citations) https://www.scbt.com/fr/p/brg-1-antibody-h-10
SETD1B (7 citations) https://www.ptglab.com/products/SETD1B-Antibody-55005-1-AP.htm
HOPX (34 citations) https://www.ptglab.com/products/HOPX-Antibody-11419-1-AP.htm
MLF2 (2 citations) https://www.ptglab.com/products/MLF2-Antibody-11835-1-AP.htm
RBM15B (12 citations) https://www.ptglab.com/products/RBM15B-Antibody-22249-1-AP.htm
DNMT1 (14 citations) https://www.activemotif.com/catalog/details/39204/dnmt1-antibody-mab
HDAC1 (248 citations) https://www.abcam.com/en-us/products/primary-antibodies/hdac1-antibody-ab7028
EZH2 (85 citations) https://www.bdbiosciences.com/en-ch/products/reagents/microscopy-imaging-reagents/immunofluorescence-reagents/purified-mouse-anti-ezh2.612666
ASH2L (1 citation) https://www.novusbio.com/products/ash2l-antibody_nb600-281

Eukaryotic cell lines

Policy information about cell lines and Sex and Gender in Research

Cell line source(s)

Mouse embryonic stem cells derived from male blastocysts of mixed 129-C57Bl/6 background (159.2 mESCs)
Human embryonic kidney cells (HEK 293T)
Mouse embryonic fibroblasts (MEF DR4)

Authentication

HEK 293T and MEF DR4 cells were ordered from ATCC. 159.2 mESCs were obtained from R. Murr (UNIGE).
For genome engineering authentication, KO and KI cell lines were confirmed by genotyping PCR, Sanger sequencing and Western blot analysis when working antibodies were available.

Mycoplasma contamination

All cell lines tested negative for mycoplasma contamination.

Commonly misidentified lines (See ICLAC register)	None

Plants

Seed stocks	*Report on the source of all seed stocks or other plant material used. If applicable, state the seed stock centre and catalogue number. If plant specimens were collected from the field, describe the collection location, date and sampling procedures.*
Novel plant genotypes	*Describe the methods by which all novel plant genotypes were produced. This includes those generated by transgenic approaches, gene editing, chemical/radiation-based mutagenesis and hybridization. For transgenic lines, describe the transformation method, the number of independent lines analyzed and the generation upon which experiments were performed. For gene-edited lines, describe the editor used, the endogenous sequence targeted for editing, the targeting guide RNA sequence (if applicable) and how the editor was applied.*
Authentication	*Describe any authentication procedures for each seed stock used or novel genotype generated. Describe any experiments used to assess the effect of a mutation and, where applicable, how potential secondary effects (e.g. second site T-DNA insertions, mosiacism, off-target gene editing) were examined.*
